# Patient-reported Quality of Life Outcomes in Patients Treated for Muscle-invasive Bladder Cancer with Radiotherapy ± Chemotherapy in the BC2001 Phase III Randomised Controlled Trial^☆^

**DOI:** 10.1016/j.eururo.2019.11.001

**Published:** 2020-02

**Authors:** Robert A. Huddart, Emma Hall, Rebecca Lewis, Nuria Porta, Malcolm Crundwell, Peter J. Jenkins, Christine Rawlings, Jean Tremlett, Leila Campani, Carey Hendron, Syed A. Hussain, Nicholas D. James, on behalf of the BC2001 Investigators

**Affiliations:** a The Institute of Cancer Research, London, UK; b Royal Devon & Exeter NHS Foundation Trust, Exeter, UK; c Gloucestershire Oncology Centre, Cheltenham General Hospital, Cheltenham, UK; d Torbay and South Devon NHS Foundation Trust, Torquay, UK; e Brighton & Sussex University Hospitals NHS Trust, Brighton, UK; f University of Birmingham, Birmingham, UK; g Academic unit of Oncology, Department of Oncology and Metabolism, Medical School, University of Sheffield, Sheffield, UK; h University Hospitals Birmingham NHS Foundation Trust, Birmingham, UK; i Royal Marsden NHS Foundation Trust, London, UK

**Keywords:** BC2001, Chemoradiotherapy, Health-related quality of life, Muscle-invasive bladder cancer, Patient-reported outcomes, Randomised controlled trial

## Abstract

**Background:**

BC2001, the largest randomised trial of bladder-sparing treatment for muscle-invasive bladder cancer, demonstrated improvement of local control and bladder cancer–specific survival from the addition of concomitant 5-fluorouracil and mitomycin C to radiotherapy.

**Objective:**

To determine the impact of treatment on the health-related quality of life (HRQoL) of BC2001 participants.

**Design, setting, and participants:**

458 UK patients with T2-T4a N0 M0 transitional cell carcinoma of the bladder.

**Intervention:**

Patients were randomised to the chemotherapy comparison (radiotherapy, 178, or chemoradiotherapy, 182); and/or to the radiotherapy comparison (standard, 108, or reduced high-dose volume radiotherapy, 111).

**Outcome measurements and statistical analysis:**

Patients completed Functional Assessment of Cancer Therapy—Bladder (FACT-BL) questionnaires at baseline, end of treatment (EoT), and 6, 12, 24, 36, 48, and 60 months after radiotherapy. The primary endpoint was change from baseline in the bladder cancer subscale (BLCS) at 12 months.

**Results and limitations:**

Data were available for 331 (92%) and 204 (93%) participants at baseline and for 192 (54%) and 114 (52%) at 12 months for the chemotherapy and radiotherapy comparisons, respectively. HRQoL declined at EoT (BLCS –5.06 [99% confidence interval: –6.12 to –4.00, *p* <  0.001]; overall FACT-B TOTAL score –8.22 [–10.76 to –5.68, *p* <  0.01]), recovering to baseline at 6 months and remaining similar to baseline subsequently. There was no significant difference between randomised groups at any time point.

**Conclusions:**

Immediately following (chemo)radiotherapy, a significant proportion of patients report declines in HRQoL, which improve to baseline after 6 months. Two-thirds of patients report stable or improved HRQoL on long-term follow-up. There is no evidence of impairment in HRQoL resulting from the addition of chemotherapy.

**Patient summary:**

Quality of life of bladder cancer patients treated with radiotherapy ± chemotherapy deteriorates during treatment, but improves to at least pretreatment levels within 6 months. Addition of chemotherapy to radiotherapy does not affect patient-reported quality of life.

## 1 Introduction

The BC2001 trial is the largest randomised trial of radiotherapy in muscle-invasive bladder cancer (MIBC) conducted to date. We previously reported that addition of chemotherapy to radiotherapy significantly improves clinical outcomes without a significant increase in clinician-reported toxicity, and that reduction of the bladder volume exposed to high-dose radiotherapy does not impact disease control or clinician-reported toxicity [1], [2]. Here, we present the 5-year, patient-reported, health-related quality of life (HRQoL) outcomes of the trial.

There are few papers describing long-term toxicity or patient-reported outcomes following bladder preservation treatment for MIBC. Previously published studies of bladder cancer patient–reported outcomes, though reassuring, are limited by being retrospective in nature, and there is a paucity of data from randomised controlled trials [3], [4], [5], [6]. We therefore planned a prospective assessment of patient-reported outcomes within BC2001, using the Functional Assessment of Cancer Therapy—Bladder (FACT-BL) questionnaire [7]. The main objective was to document the longitudinal quality of life experience of patients undergoing radical radiotherapy and to compare it between randomised treatment groups.

## 2 Patients and methods

### 2.1 Trial design and participants

BC2001 is a phase III trial with a partial 2 × 2 factorial design conducted at 45 UK hospitals. Patients with localised MIBC were randomised 1:1 to (1) the chemotherapy comparison, to receive radiotherapy with chemotherapy (cRT) or without chemotherapy (RT), and could also be randomised to (2) the radiotherapy comparison, to receive standard whole-bladder radiotherapy (stRT) or reduced high-dose volume radiotherapy (RHDVRT) with tumour boost; see Supplementary Fig. 1 for trial design. Recruitment to both randomisations was encouraged but optional according to patient eligibility and preference. All participants received conformal radiotherapy on consecutive weekdays according to the local hospital’s standard regimen (either 55 Gy/20 fractions or 64 Gy/32 fractions). Patients randomised to chemotherapy received intravenous mitomycin C (12 mg/m^2^) on day 1 of radiotherapy and continuous infusion of 5-fluorouracil (5-FU) 500 mg/m^2^/24 h for 5 days during radiotherapy fractions 1–5 and 16–20. Full details have been reported previously [1], [2].

The trial was registered (ISRCTN68324339), approved by the North West Multicentre Research Ethics Committee (00/8/075), sponsored by the University of Birmingham, and conducted in accordance with the principles of good clinical practice. All the participants provided written informed consent. The Cancer Research UK Clinical Trials Unit at the University of Birmingham and the Clinical Trials and Statistics Unit at the Institute of Cancer Research (ICR-CTSU; London, UK) shared study coordination and data management. The ICR-CTSU conducted central statistical data monitoring and statistical analyses.

### 2.2 HRQoL study

All BC2001 participants were asked to consent to the optional HRQoL study. Questionnaires were completed in clinic on paper at baseline, at the end of treatment, at 6 months from the end of treatment, and then annually to 5 years.

The FACT-BL questionnaire incorporates 39 items with five-point Likert scale answers and five subscales: physical well-being (PWB), social well-being (SWB), emotional well-being (EWB), functional well-being (FWB), and bladder cancer subscale (BLCS) [7]. For questions phrased negatively, scoring was reversed, so high scores are indicative of better HRQoL throughout. Scoring and management of missing items were dealt with in accordance with the FACIT administration and scoring guidelines [8]. See the Supplementary material (Appendix 1) for further details.

### 2.3 Outcomes

The primary endpoint for both the chemotherapy and radiotherapy comparisons was the change from baseline score in BLCS. The primary time point of interest was 1 year.

Secondary endpoints included changes from baseline in (1) FACT-BL total score (TOTAL), generated as the sum of all items in the FACT-BL questionnaire; (2) separate component subscales (PWB, SWB, EWB, and FWB); (3) the Trial Outcome Index (TOI) score (sum of PWB subscale, FWB subscale, and BLCS); and (4) specific items within the BLCS relating to urinary function, bowel function, and male sexual function.

Two exploratory analyses were conducted: firstly, we explored the impact of treatment on HRQoL at an individual level, through a comparison of the percentage of participants experiencing a minimal clinically significant positive or negative change from baseline. This was defined as a three-point change in BLCS, a five-point change in TOI, and a seven-point change in TOTAL score, with thresholds based on previous work on FACT questionnaires [9], [10], [11]. Secondly, the effect of pretreatment with neoadjuvant chemotherapy was also explored in a nonrandomised comparison between patients pretreated and those not pretreated.

### 2.4 Statistical analysis

BC2001 was powered around the primary endpoint of locoregional control; HRQoL analyses were prospectively planned as a substudy of the trial.

Analyses were performed for the (1) overall trial population, (2) chemotherapy comparison population, and (3) radiotherapy comparison population. All analyses were performed on the intention-to-treat population, with sensitivity analyses to assess robustness of the results. All analyses were conducted with Stata version 13.1 [12].

For each randomised comparison, analysis of covariance (ANCOVA) regression models were used to formally test for a difference in the mean change from baseline, after adjusting for alternate randomisation, radiotherapy fractionation, and baseline score. Only patients with paired baseline and follow-up data were included in the analysis. A significance level of 5% was used to test for the principal outcome measure (difference in BLCS mean between treatment groups within each comparison at 1 year, with 95% confidence interval [CI] for the estimated difference). Interaction between the two randomised interventions was tested using an ANCOVA model fitted in patients included in both randomisations. A 1% significance level (and corresponding 99% CI) was used for all other endpoints to make allowance for multiplicity in testing.

See the Supplementary material (Appendix 1) for further details on study methods.

## 3 Results

### 3.1 Patients

Between August 2001 and April 2008, 458 BC2001 participants were recruited from 45 UK centres; 452 completed at least one HRQoL assessment. Of these, 216 patients entered the radiotherapy comparison (107 stRT/109 RHDVRT) and 355 entered the chemotherapy comparison (179 cRT/176 RT). A total of 119 patients entered both comparisons. At 1 year, 114 (53%) and 192 (54%) patients in the radiotherapy and chemotherapy comparisons, respectively, provided HRQoL data; this represented, respectively, 68% and 70% of expected questionnaires (Supplementary Table 1). Amongst patients without 1-year or later HRQoL questionnaires, 55% had an invasive recurrence or cystectomy before 12 months, while amongst patients with completed 1-year questionnaires, only 11% had an invasive event before 12 months (Supplementary Fig. 2). Baseline features of patients with and without HRQoL data at 1 year are given in Supplementary Table 2. Patients without HRQoL data were similar to those with data apart from a greater frequency of residual mass and incomplete resection at baseline.

### 3.2 Change from baseline scores

#### 3.2.1 Overall trial population

Table 1 describes the median and quartile scores for each subscale in the FACT-BL at baseline, end of treatment, 1 year, and 5 years.

**Table 1:** General FACT-BL scores per subscale and time point—overall population

	Baseline			EoT			1 year			5 years		
	*N*	Median	Q1–Q3	*N*	Median	Q1–Q3	*N*	Median	Q1–Q3	*N*	Median	Q1–Q3
BLCS	421	34	29–39	349	29	24–35	242	35	30–37	109	34	31–38
TOTAL	421	124	108–133	349	115	97–130	240	126	113–137	107	127	116–136
TOI	418	80	70–88	346	71	57–83	240	82	72–90	107	83	74–89
EWB	420	20	17–22	352	21	19–23	242	21	19–23	108	22	20–24
FWB	421	21	17–25	350	19	14–24	242	22	17–26	109	22	18–26
SWB	415	25	22–28	345	25	21–27	242	25	21–28	105	24	20–27
PWB	423	25	22–27	351	24	20–26	242	26	23–27	108	26	24–27

BLCS = bladder cancer subscale; EoT = end of treatment; EWB = emotional well-being; FACT-BL = Functional Assessment of Cancer Therapy—Bladder; FWB = functional well-being; PWB = physical well-being; Q1 = 1st quartile (25% percentile); Q3 = 3rd quartile (75% percentile); SWB = social well-being; TOI = Trial Outcome Index; TOTAL = FACT-BL total score.

**Fig. 1:**
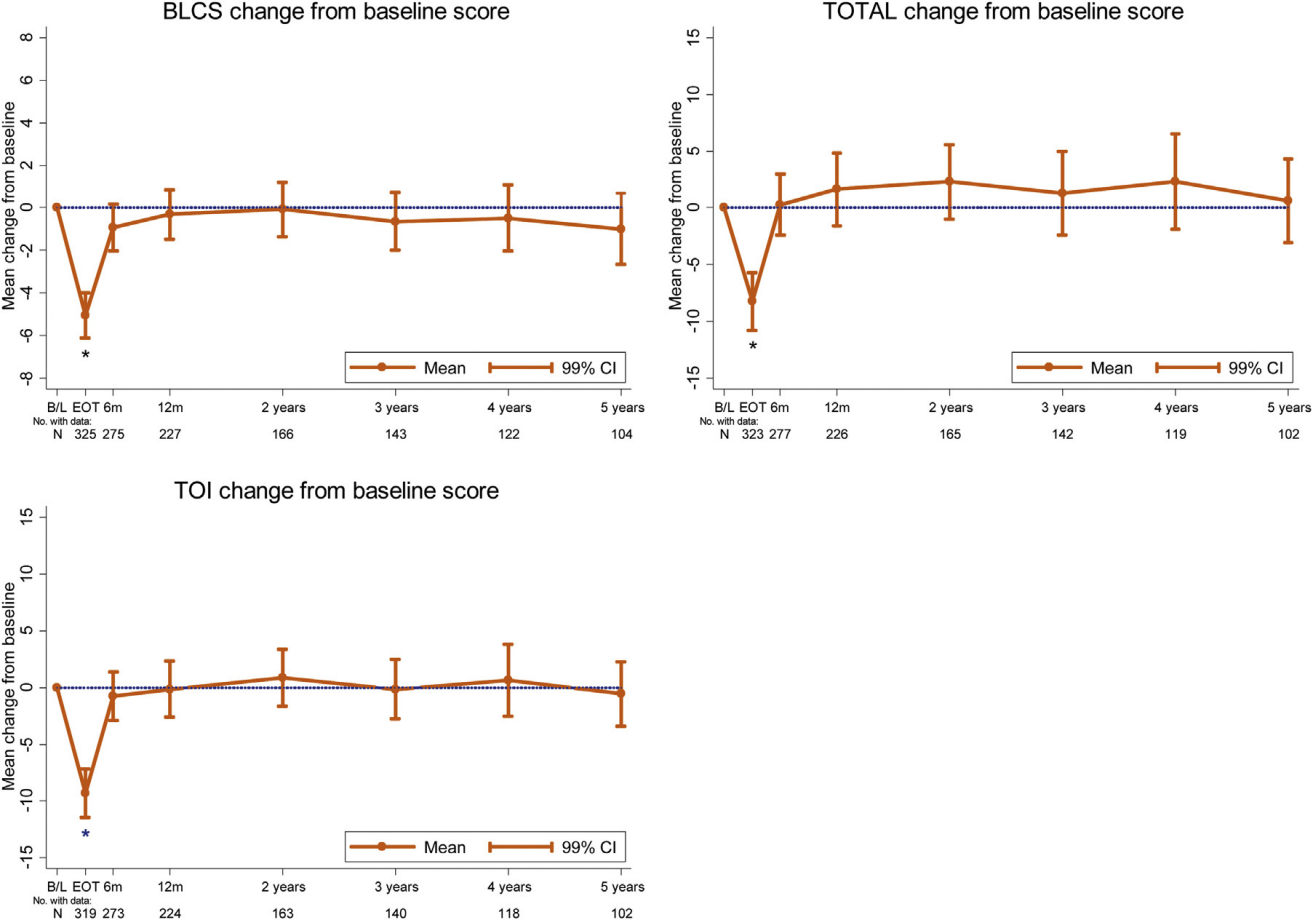
Mean change from baseline (with 99% confidence intervals) in FACT-BL BLCS, TOI, and TOTAL scores for overall population. B/L = baseline; BLCS = bladder cancer subscale; CI = confidence interval; EoT = end of treatment; FACT-BL = Functional Assessment of Cancer Therapy—Bladder; TOI = Trial Outcome Index; TOTAL = FACT-BL total score.

For the whole BC2001 population, a fall in HRQoL is seen immediately following radiotherapy in the majority of domains (Fig. 1 and Supplementary Fig. 3). There is a mean change in BLCS score of –5.06 (99% CI: –6.12 to –4.00; *p* <  0.001), a mean change in the TOTAL score of –8.22 (99% CI: –10.76 to –5.68; *p* <  0.001), and a mean change in the TOI score of –9.34% (99% CI: –11.47 to –7.20; *p* <  0.001). By 6 months after radiotherapy, HRQoL scores have improved and returned to baseline levels.

By 1 year, all subscales have recovered to at least baseline levels (*p* >  0.01), and this is maintained or improved on subsequent follow-up. The EWB score significantly improves above baseline at the end of treatment, with a mean change of 1.14 (99% CI: 0.68 to 1.61; *p* <  0.001), and remains above baseline throughout follow-up.

#### 3.2.2 Chemotherapy comparison

**Fig. 2:**
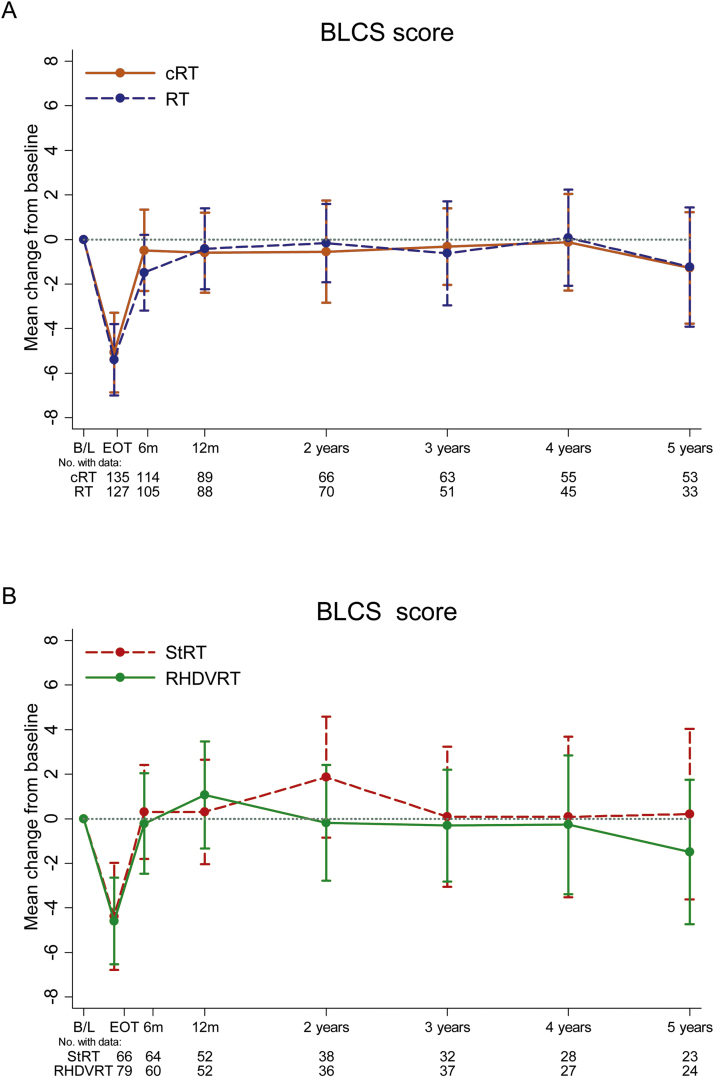
Mean change from baseline (with 99% confidence intervals) in BLCS score in the treatment comparisons: (A) chemotherapy comparison and (B) radiotherapy comparison. B/L = baseline; BLCS = bladder cancer subscale; cRT = radiotherapy with chemotherapy; EoT = end of treatment; RHDVRT = reduced high-dose volume radiotherapy; RT = radiotherapy; StRT = standard whole-bladder radiotherapy.

The primary endpoint for the chemotherapy comparison is shown in Fig. 2A . Table 2 provides the treatment differences at 1 year for change from baseline in all subscales. For BLCS, the adjusted difference between cRT and RT at 1 year was 0.18 (95% CI: –1.60 to 1.96; *p* =  0.8). No significant differences were found in any subscale or at any time point (Supplementary Fig. 4).

**Table 2:** Change from baseline at 1 year for the randomised comparisons

	cRT			RT			Difference cRT – RT^a^		
1 yr	*N*	Mean^b^	99% CI	*N*	Mean^b^	99% CI	Mean^c^	99% CI^d^	*p* value
BLCS	89	–0.59	–2.42, 1.24	88	–0.42	–2.27, 1.44	0.18	–1.60, 1.96^d^	0.8
TOTAL	89	0.67	–4.56, 5.89	86	2.67	–2.46, 7.80	–1.79	–8.3, 4.72	0.5
TOI	88	–0.50	–4.45, 3.45	86	0.94	–3.08, 4.96	–1.07	–6.21, 4.07	0.6
EWB	91	0.90	–0.26, 2.06	88	1.48	0.46, 2.51	–0.47	–1.68, 0.75	0.3
FWB	89	0.25	–1.54, 2.04	88	0.99	–0.69, 2.67	–0.67	–2.77, 1.44	0.4
SWB	88	–0.16	–1.80, 1.47	88	0.22	–1.12, 1.55	–0.30	–2.07, 1.48	0.7
PWB	91	–0.11	–1.33, 1.10	88	0.38	–0.89, 1.66	–0.54	–2.14, 1.07	0.4

	stRT			RHDVRT			Difference stRT – RHDVRT^a^		
	*N*	Mean^b^	99% CI	*N*	Meanb	99% CI	Mean^c^	99% CI^d^	*p* value
BLCS	52	0.31	–2.13, 2.74	52	1.06	–1.43, 3.55	2.01	–0.31, 4.33^d^	0.089
TOTAL	53	2.09	–5.58, 9.75	53	5.71	–0.66, 12.07	6.29	–2.54, 15.12	0.064
TOI	52	0.01	–5.96, 5.98	52	1.79	–2.79, 6.38	4.05	–2.75, 10.85	0.12
EWB	54	1.27	–0.22, 2.76	52	1.98	0.78, 3.18	0.86	–0.52, 2.24	0.11
FWB	53	–0.12	–3.19, 2.94	53	0.26	–1.61, 2.14	1.21	–1.98, 4.39	0.3
SWB	53	0.21	–2.34, 2.76	51	0.41	–1.33, 2.16	1.21	–1.60, 4.02	0.3
PWB	54	–0.19	–2.13, 1.75	53	0.69	–0.54, 1.91	1.16	–0.84, 3.16	0.13

ANCOVA = analysis of variance; BLCS = bladder cancer subscale; CI = confidence interval; cRT = radiotherapy with chemotherapy; EWB = emotional well-being; FWB = functional well-being; PWB = physical well-being; RHDVRT = reduced high-dose volume radiotherapy; RT = radiotherapy without chemotherapy; stRT = standard whole-bladder radiotherapy; SWB = social well-being; TOI = Trial Outcome Index; TOTAL = FACT-BL total score.

a Positive differences favour experimental group (cRT or RHDVRT, respectively).

b Mean change from baseline at 1 year, within each group.

c Mean difference between groups, computed by ANCOVA and adjusted by alternate randomisation, radiotherapy fractionation schedule, and baseline score.

d 95% CI for comparison of primary endpoint change in BLCS at 1 year.

#### 3.2.3 Radiotherapy comparison

For the radiotherapy comparison reported in Fig. 2B and Table 2, the difference between stRT and RHDVRT at 1 year in change from baseline BLCS score is 2.0 (95% CI: –0.31 to 4.33; *p* =  0.089). No significant differences were found in any subscale or at any time point (Supplementary Fig. 5).

For patients included in both randomisations, there was no evidence of an interaction effect between the two randomised interventions in change in BLCS (*p* =  0.3), TOTAL score (*p* =  0.3), or TOI (*p* =  0.4) at 1 year.

#### 3.2.4 Pretreated patients

No detrimental impact on HRQoL was observed in those patients who had received neoadjuvant chemotherapy prior to trial entry (Supplementary Tables 3 and 4, and Supplementary Fig. 6). Baseline scores were similar between the patients who received neoadjuvant chemotherapy and those who did not. There was no statistically significant difference in change from baseline BLCS (0.93; 99% CI: –1.39 to 3.25; *p* =  0.3) or TOTAL (6.17; 99% CI: –0.50 to 12.85; *p* =  0.017) subscales, though the estimated difference in the TOTAL score favoured patients who had received neoadjuvant chemotherapy compared with those who had no prior therapy.

### 3.3 Individual items on the BLCS

**Fig. 3:**
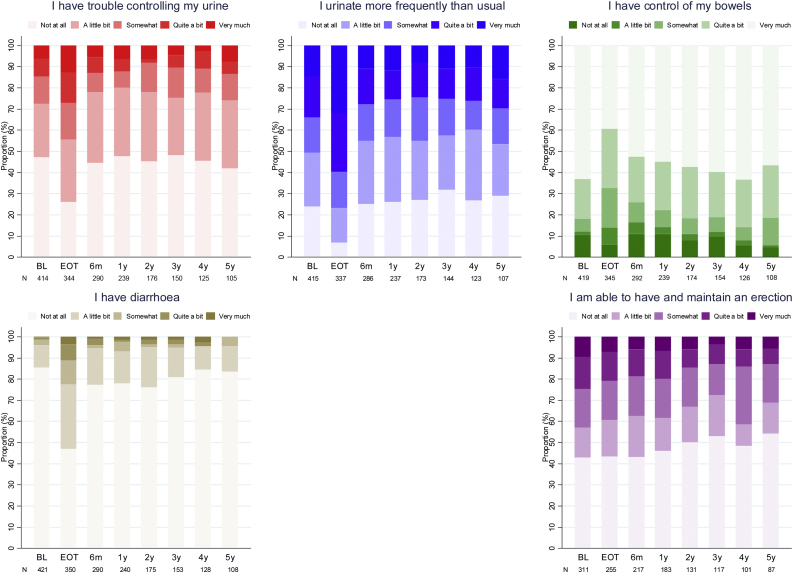
Individual items in the bladder cancer subscale of FACT-BL. BL = baseline; EoT = end of treatment; FACT-BL = Functional Assessment of Cancer Therapy—Bladder.

At 1 year, the percentage of participants stating that they had “quite a bit” or “very much” trouble controlling their urine was 12% (baseline 15%), urinating more than usual was 25% (baseline 34%), not having control of their bowels was 14% (baseline 12%), and having diarrhoea was 2.1% (baseline 1.2%). Amongst males, the percentage being able to have or maintain an erection (“quite a bit” or “very much”) was 20% (baseline 24%). Fig. 3 reports the percentages for all items and all time points in the overall population. Similar proportions were found in the treatment arms of the randomised comparisons (Supplementary Fig. 7).

### 3.4 Impact of treatment on individuals

**Fig. 4:**
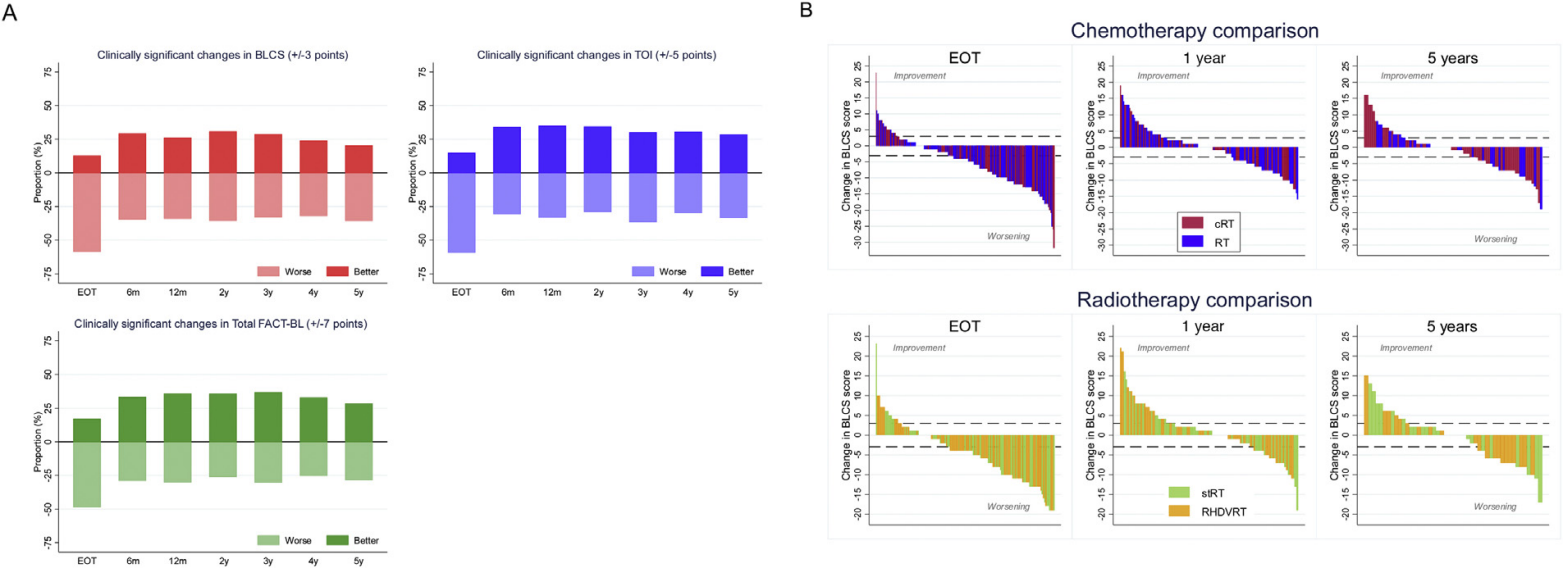
Impact of treatment on individuals. (A) Proportion of patients with clinically significant changes in BLCS, TOI, and TOTAL—overall population. Clinically significant changes are defined as changes from baseline above (better) or below (worse) the subscale threshold (BLCS ± 3 points, TOI ± 5 points, TOTAL ± 7 points) at each time point. Patients without clinically significant changes are not shown in the figure. (B) Waterfall plot of the absolute change in BLCS score from baseline at each time point, per randomised comparison. Each bar represents a patient. The dashed line represents clinical significance (±3). BLCS = bladder cancer subscale; cRT = radiotherapy with chemotherapy; EoT = end of treatment; FACT-BL = Functional Assessment of Cancer Therapy—Bladder; RHDVRT = reduced high-dose volume radiotherapy; RT = radiotherapy without chemotherapy; StRT = standard whole-bladder radiotherapy; TOI = Trial Outcome Index; TOTAL = FACT-BL total score.

As expected from the mean scores, in the overall population, a substantial proportion of participants reported worsening of HRQoL at the end of treatment (Fig. 4A ). On the BLCS score, 59% reported worsening of symptoms at the end of treatment, similar to the pattern seen with the TOI score, while 49% reported worsening symptoms in the TOTAL score. Improved HRQoL was reported by 13%, 15%, and 17% participants on the BLCS, TOI, and TOTAL scores, respectively.

By 6 months, the proportions reporting worsening had fallen to 35%, 30%, and 29% in the BLCS, TOI and TOTAL scores, respectively, with improvement reported in 29%, 34%, and 33%, respectively. On further follow-up, these relative proportions remain fairly constant, with between 32% and 36% of patients reporting worsening on the BLCS score (Fig. 4B), 28% and 36% on the TOI score, and 25% and 30% on the TOTAL score, with similar numbers reporting improvement.

No significant difference was found between randomised groups within each comparison in the proportions of patients reporting clinically relevant improvement/worsening on any scale or at any time point (Supplementary Tables 5–10).

For all endpoints, the preplanned sensitivity analyses confirmed the results reported (results not shown).

## 4 Discussion

We report the largest study of patient-reported outcomes after bladder-preserving treatment for MIBC. A key strength is that these are, to our knowledge, the only such data prospectively collected within a randomised controlled trial. A drawback, however, is that not all patients returned HRQoL questionnaires at the key defined time point, most frequently due to prior recurrence or cystectomy, so it is likely that the impact of recurrence is not fully captured in these data. However, approximately 70% of patients returned questionnaires at 1 year, which is similar to the 77% return rate observed in the cohort HRQoL study of Mak et al [13]. Response rates declined further over time, though ˜60% of expected questionnaires were received at 5 years. It is likely that comorbidities and competing risks (including death) would contribute to nonresponse in this elderly population.

The results, as expected, show an early reduction in HRQoL at the end of treatment consistent with treatment-related toxicity; by 6 months, this has largely improved so that mean scores return to baseline. The addition of chemotherapy to radiotherapy did not affect the reported HRQoL outcomes, providing evidence that the large beneficial effect on locoregional control with chemoradiotherapy using 5-FU and mitomycin C is achieved without an appreciable adverse impact on HRQoL. Likewise, there is no evidence that neoadjuvant chemotherapy impairs HRQoL. If anything, there was a trend towards improved HRQoL in these patients, though as baseline is taken after neoadjuvant treatment, this may have impaired baseline scores and improvement could represent recovery from neoadjuvant chemotherapy-related toxicity. Alternatively, the debulking effect of the prior chemotherapy may have left a better functioning bladder than one that has recently undergone transurethral resection of bladder tumour, resulting in improved radiation tolerance.

These results are in line with the limited previous data available. Lagrange et al [14] investigated HRQoL using the EORTC QLQ-C30 tool in 51 patients in a phase II nonrandomised trial of 5-FU and cisplatin given during whole pelvic radiotherapy. Similarly to our study, they demonstrated a fall in HRQoL immediately after treatment, which slightly improved above baseline across subscales after 6 months. This is supported by other smaller studies that report long-term HRQoL outcomes similar to population averages [4], [5], [6], [15]. The exception to this was emotional wellbeing, which improved at the end of treatment and beyond, perhaps emphasising the positive psychological impact of receiving radical treatment and the support of care providers, despite potential side effects.

Analysis of clinically relevant changes in HRQoL on an individual basis perhaps provides a richer understanding of the impact of treatment on patients. It is clear from this analysis that the majority of patients experience some deterioration in symptoms at the end of radiotherapy, though many subsequently recover, and a substantial proportion of patients have HRQoL similar to, or better than, baseline over subsequent follow-up. The observation of a mean overall score similar to baseline is thus achieved, as roughly similar numbers of patients have improvement and deterioration in quality of life. Similar findings have been shown in previous studies. For example, Zietman et al [4] reported in a retrospective study of 48 patients treated with bladder-conserving therapy that patients had similar or higher than population average scores. However, seven had reduced bladder compliance, two reported bladder hypersensitivity, and seven were distressed by bowel urgency. Likewise, Henningsohn et al [3] reported that 74% of patients had “little or no” urinary distress in a cohort study of 58 patients after radiotherapy. In this study, 32% of radiotherapy patients had some gastrointestinal symptoms compared with 9% of population controls. Lagrange et al [14] again reported that over 70% had “satisfactory” urinary function after 6 months—a similar proportion to our study.

We can thus conclude from these data that though most patients can expect satisfactory HRQoL after radiotherapy, a subgroup will have a decline. Understanding the drivers of this deterioration should be a priority in future HRQoL research in bladder cancer. Can those patients likely to experience a persistent decline in HRQoL be predicted from baseline characteristics, tumour-related characteristics, prior therapies (eg, BCG for prior non muscle invasive bladder cancer), or even their baseline germline genetics? Recently, a number of common genetic variants have been associated with toxicity in prostate and breast radiotherapy [16]; a study in bladder cancer patients would be of interest. We also need to consider whether there are any interventions that can be undertaken to moderate the impact of radiotherapy treatment on HRQoL. For instance, there are limited data suggesting that instillation of hyaluronic acid may reduce radiation cystitis in gynaecological patients [17].

It is disappointing that we could not demonstrate any evidence of symptomatic or HRQoL improvement in the group of patients treated with a modified radiotherapy volume. This treatment approach was developed on the basis that restricting the high-dose volume may reduce the bladder shrinkage/fibrosis sometimes seen with whole-bladder radiotherapy. This was supported by retrospective studies suggesting that global bladder radiation tolerance was less than focal tolerance [18]. Our study of this issue was undermined by early closure of the radiotherapy comparison randomisation due to slow recruitment and a substantial number of protocol violations, resulting in low power to assess treatment effects. Additionally, this study was performed in the era before intensity-modulated and image-guided radiotherapy, with the use of large margins around the tumour so the amount of bladder sparing will have been limited. Based on a successful pilot study [19], we are currently revisiting this issue in conjunction with dose escalation in a randomised phase II trial (RAIDER ISRCTN26779187).

Chemoradiotherapy is a widely used bladder-conserving alternative to radical cystectomy. This study does not directly address the relative HRQoL benefits of surgery and (chemo)radiotherapy. Indeed, given the very different treatment modalities, making such a comparison is challenging; for instance, a patient who has a radical cystectomy and uteroileal bypass does not pass urine and therefore reporting of urinary symptoms has a very different interpretation. Several retrospective or cohort studies suggest that HRQoL after radiotherapy is at least equivalent and possibly superior to that following cystectomy [5], [20], [21], [22], [23], [24]. The largest of these is a cohort study of 173 survivors of bladder cancer 7–9 years after cystectomy or chemoradiotherapy, which reported better HRQoL in most domains after chemoradiotherapy, though this study is limited by a lack of baseline data [13]. One aspect of this study was better sexual function after chemoradiotherapy, which is supported by our data that show, at worst, a modest decline in sexual function after radiotherapy with or without chemotherapy. We previously published a small randomised feasibility study of selective bladder preservation and radical cystectomy (SPARE) [15]. The patients who had radiotherapy had similar HRQoL scores to those reported here, which, though not statistically significant, tended to be higher than those achieved by patients having cystectomy.

## 5 Conclusions

This study shows that overall, after an initial fall immediately after treatment, HRQoL recovers to baseline levels and is maintained at this level to 5 years for bladder cancer patients treated with radiotherapy. Addition of concomitant chemotherapy or use of neoadjuvant chemotherapy has no significant impact on HRQoL, further supporting the routine use of 5-FU and mitomycin C in this setting. Although the majority of patients maintain HRQoL similar to or better than baseline, around one-third experience some persistent detriment. This has to be considered in the context of the significant quality of life impact of the alternative treatment of cystectomy.

  ***Author contributions*:** Robert A. Huddart and Emma Hall had full access to all the data in the study and takes responsibility for the integrity of the data and the accuracy of the data analysis.

*Study concept and design*: Huddart, Hall, Hussain, James.

*Acquisition of data*: Lewis, Hendron.

*Analysis and interpretation of data*: Huddart, Hall, Lewis, Porta, Crundwell, Jenkins, Rawlings, Tremlett, Hendron, Hussain, James.

*Drafting of the manuscript*: Huddart, Hall, Lewis, Crundwell, Jenkins, Rawlings, Tremlett, Hendron, Hussain, James.

*Critical revision of the manuscript for important intellectual content*: Huddart, Hall, Lewis, Crundwell, Jenkins, Rawlings, Tremlett, Hendron, Campani, Hussain, James.

*Statistical analysis*: Hall, Porta.

*Obtaining funding*: Huddart, Hall, Hussain, James.

*Administrative, technical, or material support*: Lewis, Hendron.

*Supervision*: Huddart, Hall, Lewis, Crundwell, Jenkins, Rawlings, Tremlett, Hendron, Hussain, James.

*Other*: None.

  ***Financial disclosures:*** Robert A. Huddart certifies that all conflicts of interest, including specific financial interests and relationships and affiliations relevant to the subject matter or materials discussed in the manuscript (eg, employment/affiliation, grants or funding, consultancies, honoraria, stock ownership or options, expert testimony, royalties, or patents filed, received, or pending), are the following: None.

  ***Funding/Support and role of the sponsor*:** BC2001 was supported by Cancer Research UK (CRUK/01/004) with programme grants to support the work of the CR UK Cancer Trials Unit, Birmingham (C547/A2606; C547/A6845; C9764/A9904) and ICR-CTSU (C1491/A9895; C1491/A15955). Trial recruitment was facilitated at participating sites by the National Institute for Health Research (NIHR)-funded National Cancer Research Network. We acknowledge NHS funding to the NIHR Biomedical Research Centre at The Royal Marsden and the ICR. The funder of the study had no role in study design, data collection, data analysis, data interpretation, or writing of the report. The coauthors had full access to all data in the study, and Robert Huddart and Emma Hall as first authors had final responsibility for the decision to submit for publication.

  ***Acknowledgements:*** Grateful thanks to all the patients who participated in this study, all involved staff at the participating centres, and trials unit staff at ICR-CTSU, including Miguel Miranda and Rachel Todd, and Birmingham CRUK-CTRU. We would also like to thank the BC2001 Trial Management Group members (past and present), and the Independent Data Monitoring Committee and Trial Steering Committee for overseeing the trial.
