# Supplementary material for: Patient-reported Quality of Life Outcomes in Patients Treated for Muscle-invasive Bladder Cancer with Radiotherapy ± Chemotherapy in the BC2001 Phase III Randomised Controlled Trial[image]
Source: Eur Urol. 2020 Feb;77(2):260–8. doi: 10.1016/j.eururo.2019.11.001 (PMC6983941; doi:10.1016/j.eururo.2019.11.001)
Supplement: Supplementary file 1 [file mmc1.docx]

**Supplementary material**

**Supplementary Figure 1. BC2001 trial schema**

*BC2001 has a partial 2x2 factorial design: Patients were permitted to enter either one or both randomisations ie the CT comparison (RT alone vs cRT) or the RT comparison (standard volume RT vs reduced high dose volume RT) or both. Treatment allocation in both comparisons was 1:1. Randomisation was stratified by centre and use of neoadjuvant chemotherapy.*

*Eligible patients had histologically confirmed T2-T4a N0 M0 MIBC, WHO performance status 0 to 2; GFR> 25ml/min. For RT comparison: single tumour at time of invasive disease diagnosis.*


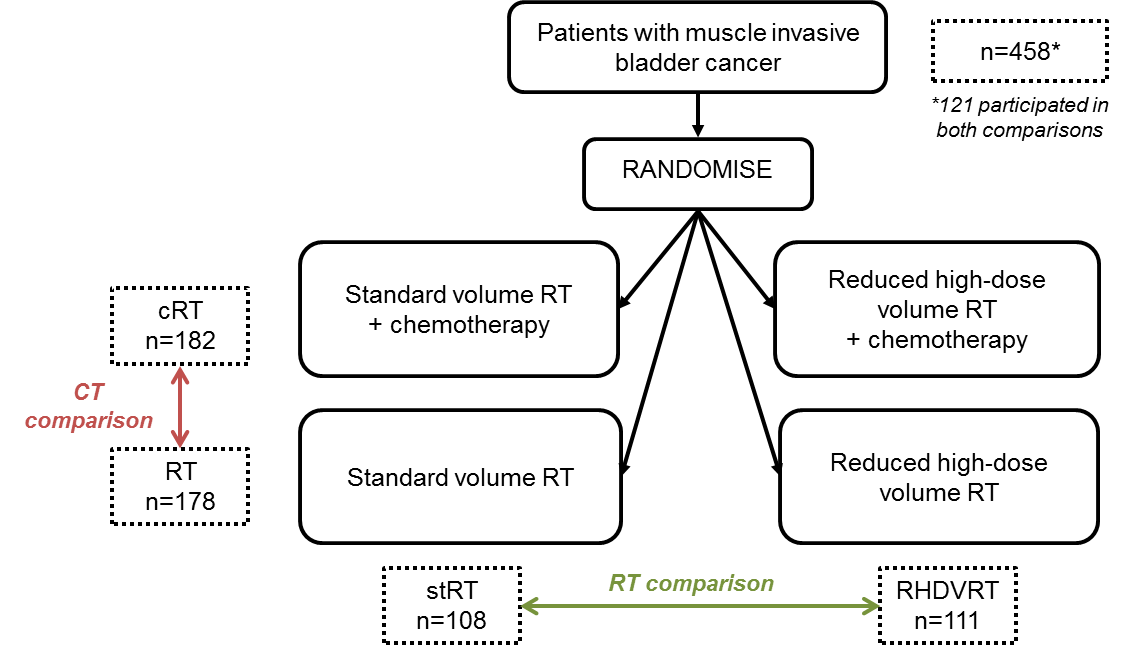


**Supplementary Appendix 1 – Further details on methodology and statistical methods**

*The FACT-BL questionnaire*

The Functional Assessment of Cancer Therapy - Bladder (FACT-BL) questionnaire consists of 39 items with five point Likert scale answers (0 - not at all, 1 - a little bit, 2 - somewhat, 3 - quite a bit and 4 - very much). There are five subscales: physical well-being (PWB, 7 items), social well-being (SWB, 7 items), emotional well-being (EWB, 6 items), functional well-being (FWB, 7 items) and the bladder cancer subscale (BLCS, 12 items). For those questions phrased negatively the scoring was reversed so that high scores are indicative of better HRQoL throughout. The maximum score per subscale, taken as the cumulative sum of the items within each subscale, is 28 points, with the exception of EWB and BLCS scoring 24 and 48 points respectively.

Missing items within each subscale were imputed as the un-weighted mean of all answered items, conditional on a minimum 50% response rate, and for the TOTAL FACT-BL scale, conditional on an 80% response rate with all component subscales having valid scores, in accordance with the FACIT Administration and Scoring Guidelines.^1^ The BLCS contained items relevant to (i) men only and (ii) those with an ostomy appliance, so these factors were taken into consideration when determining the minimum response rate required for imputation to this subscale.

At the time when the statistical analysis was planned, there were no published minimally important differences for FACT-BL. There has been detailed work by Cella and colleagues (2002)^2^ who have used both distribution and anchor methodologies to determine these figures across FACIT questionnaires with reasonable consistency in values used. Following this, for the non-bladder specific score of the FACT-BL (FACT-G: PWB+FWB+EWB+SWB scores) a minimally important difference of 4.0 was proposed. Karvinen et al (2007)^3^ used FACT-BL questionnaire to assess the impact of exercise in bladder cancer patients, and referred that minimally important differences for other FACT scales were typically around 7 points^4^. We therefore set the minimally important difference for FACT-BL TOTAL score at 3 points, and as FACT-G’s minimally importance is 4, the minimally important difference for the specific BLCS score was set to 3. For the TOI score, as it contains BLCS and the functional and physical scales, the minimally important difference was set at 5 points.

*Statistical methods:*

The trial was powered around the primary endpoint of loco-regional control. For HRQoL analyses, post-hoc power calculations based on available data were done before any analysis occurred. We assumed a 5% significance level and standard deviation of seven points for the change from baseline in BLCS at one year (estimated from the whole trial population). For the chemotherapy comparison, 177 patients with paired BLCS data at baseline and one year provide 80% power to detect differences of 2.94 or greater between cRT and RT in mean change from baseline in BLCS at one year. For the radiotherapy comparison, 104 patients with paired data provide 80% power to detect differences of 3.85 or greater between stRT and RHDVRT.

Analyses were performed for 1) the overall trial population 2) the chemotherapy comparison population, and 3) the radiotherapy comparison population.

All endpoints were summarised descriptively at each timepoint, overall and by randomised group, and their evolution with time graphically represented. Summary statistics included number of observations per endpoint, timepoint and group of analysis; medians and interquartile ranges for raw scores; means and their 99% confidence intervals (CI) for changes from baseline endpoints; and frequencies and percentages for categorical endpoints.

For the overall population, mean changes from baseline were compared using two-sided paired t-tests. For the randomised comparisons, ANCOVA regression models were used to formally test for a difference in mean change from baseline, after adjusting for alternate randomisation, radiotherapy fractionation and baseline score. Only patients with paired baseline and follow-up data were included in the analysis.

A significance level of 5% was used to test for the principal outcome measure (difference in BLCS mean between treatment groups within each comparison at one year, with 95% CI for the estimated difference). A 1% significance level (and corresponding 99% CI) was used for all other endpoints to make allowance for multiplicity in testing.

All analyses were performed on the intention to treat population, and repeated in the treatment received population to assess the robustness of the results. Further sensitivity analyses included: (i) excluding any subscales containing a transformed score of 0/1 (poorest outcome) reported three months or less before a first recurrence or bladder cancer death or after a second primary cancer diagnosis, (ii) excluding any patients with less than two years of follow-up and (iii) excluding any patients with major protocol violations (per protocol population). All analyses were done with Stata version 13.1.

Individual BLCS items were descriptively summarised using frequencies and proportions at each time point. The frequency and proportion of patients experiencing a clinically relevant improvement, worsening or no difference in HRQoL at each timepoint relative to baseline was tabulated and compared between groups using Chi-squared or Fisher’s exact test as appropriate, under two degrees of freedom.

Similar analyses were performed to compare HRQoL TOTAL and BLCS scores between patients pre-treated with neo-adjuvant chemotherapy and patients who did not. A 1% significance level (and corresponding 99% CI) was also used for this exploratory analysis.

The trial was powered around the primary endpoint of loco-regional control. For HRQoL analyses, power calculations were done retrospectively but before any analysis occurred. We assumed a significance level of 5% and a standard deviation for the change from baseline in BLCS at one year of seven points, estimated from the whole trial population. For the chemotherapy comparison, the 177 patients with paired BLCS data at baseline and one year provide 80% power to detect differences of 2·94 or greater between cRT and RT in the change from baseline in BLCS means at one year. For the radiotherapy comparison, the 104 patients with paired data provide 80% power to detect differences of 3·85 or greater between stRT and RHDVRT. The power to detect the minimal clinically relevant difference of three or greater between groups in change from baseline BLCS at one year is 81% for the chemotherapy comparison and 59% for the radiotherapy comparison.

**References**

1. FACIT.org. FACIT Administration and Scoring Guidelines. In.

2. Cella D, Eton DT, Lai JS, Peterman AH, Merkel DE. Combining anchor and distribution-based methods to derive minimal clinically important differences on the Functional Assessment of Cancer Therapy (FACT) anemia and fatigue scales. *J Pain Symptom Manage.* 2002;24(6):547-561.

3. Karvinen KH, Courneya KS, North S, Venner P. Associations between exercise and quality of life in bladder cancer survivors: a population-based study. *Cancer Epidemiol Biomarkers Prev.* 2007;16(5):984-990.

4. Yost KJ, Cella D, Chawla A, et al. Minimally important differences were estimated for the Functional Assessment of Cancer Therapy-Colorectal (FACT-C) instrument using a combination of distribution- and anchor-based approaches. *J Clin Epidemiol.* 2005;58(12):1241-1251.

**Supplementary Table 1. HRQoL sub-study – questionnaire response rates**

|  | **CT comparison** | | | | | **RT comparison** | | | |
| --- | --- | --- | --- | --- | --- | --- | --- | --- | --- |
|  | **No CT** | | **CT** | |  | **stRT** | | **RHDVRT** | |
|  | **N** | **%** | **N** | **%** |  | **N** | **%** | **N** | **%** |
| **Total patients consented to QoL** | **176** | **100.0%** | **179** | **100.0%** |  | **107** | **100.0%** | **109** | **100.0%** |
| Baseline |  |  |  |  |  |  |  |  |  |
| Expected | 176 | 100.0% | 179 | 100.0% |  | 107 | 100.0% | 109 | 100.0% |
| Available | 160 | 90.9% | 171 | 95.5% |  | 101 | 94.4% | 103 | 94.5% |
| End of Treatment |  |  |  |  |  |  |  |  |  |
| Expected | 171 | | 176 | 100.0% |  | 107 | 106.0% | 106 | 100.0% |
| Available | 143 | 83.6% | 144 | 81.8% |  | 74 | 69.2% | 84 | 79.2% |
| Paired BLCS BL-EoT | 127 | 74.3% | 135 | 76.7% |  | 66 | 61.7% | 79 | 74.5% |
| Month 6 |  |  |  |  |  |  |  |  |  |
| Expected | 162 | 100.0% | 155 | 100.0% |  | 98 | 100.0% | 94 | 100.0% |
| Available | 116 | 71.6% | 121 | 78.1% |  | 69 | 70.4% | 66 | 70.2% |
| Paired BLCS BL-M6 | 105 | 64.8% | 114 | 73.5% |  | 64 | 65.3% | 60 | 63.8% |
| Month 12 |  |  |  |  |  |  |  |  |  |
| Expected | 137 | 100.0% | 139 | 100.0% |  | 84 | 100.0% | 83 | 100.0% |
| Available | 97 | 70.8% | 95 | 68.3% |  | 57 | 67.9% | 57 | 68.7% |
| Paired BLCS BL-M12 | 88 | 64.2% | 89 | 64.0% |  | 52 | 61.9% | 52 | 62.7% |
| Year 2 |  |  |  |  |  |  |  |  |  |
| Expected | 100 | 100.0% | 105 | 100.0% |  | 61 | 100.0% | 66 | 100.0% |
| Available | 75 | 75.0% | 71 | 67.6% |  | 40 | 65.6% | 40 | 60.6% |
| Paired BLCS BL-Y2 | 70 | 70.0% | 66 | 62.9% |  | 38 | 62.3% | 36 | 54.5% |
| Year 3 |  |  |  |  |  |  |  |  |  |
| Expected | 87 | 100.0% | 96 | 100.0% |  | 48 | 100.0% | 56 | 100.0% |
| Available | 57 | 65.5% | 66 | 68.8% |  | 33 | 68.8% | 41 | 73.2% |
| Paired BLCS BL-Y3 | 51 | 58.6% | 63 | 65.6% |  | 32 | 66.7% | 37 | 66.1% |
| Year 4 |  |  |  |  |  |  |  |  |  |
| Expected | 72 | 100.0% | 87 | 100.0% |  | 42 | 100.0% | 50 | 100.0% |
| Available | 46 | 63.9% | 58 | 66.7% |  | 29 | 69.0% | 31 | 62.0% |
| Paired BLCS BL-Y4 | 45 | 62.5% | 55 | 63.2% |  | 28 | 66.7% | 27 | 54.0% |
| Year 5 |  |  |  |  |  |  |  |  |  |
| Expected | 68 | 100.0% | 84 | 100.0% |  | 38 | 100.0% | 44 | 100.0% |
| Available | 36 | 52.9% | 56 | 66.7% |  | 24 | 63.2% | 26 | 59.1% |
| Paired BLCS BL-Y5 | 33 | 48.5% | 53 | 63.1% |  | 23 | 60.5% | 24 | 54.5% |

**Supplementary Figure 2. CONSORT flow diagram for BC2001 QoL sub-study – detailed**

At year 1, 351 patients overall were expected to complete a questionnaire, but 106 had a missing 1 year questionnaire: 26 had the 1-year visit missing, but had other visits afterwards (2,3,4, or 5 years); for 80 patients, no further questionnaires were received.

In these 80 patients, 44 (55%) had an invasive loco-regional recurrence, distant recurrence or cystectomy before 12 months. In the 245 patients for whom 1-year visit was available, 29 (11.8%) had an invasive loco-regional recurrence, distant recurrence or cystectomy before 12 months.There is more missing 1-year data when ‘invasive’ disease-related events occur before 12 months (p<0.001).

In the CT comparison, patients with an invasive event before 12 months and 1-year and later missing data in the non CT group represented 67% vs 50% CT group (p=0.30); patients with no missing 1-year data and events before 12 months represented 14% no CT vs 11% CT (p=0.51).

In the RT comparison, patients with an invasive event before 12 months and 1-year and later missing data represented 58% stRT vs 44% RHDVRT (p=0.54); patients with no missing 1-year data and events before 12 months represented 11% stRT vs 7% RHDVRT (p=0.74).

Though differences between treatment groups in % of an invasive event before 12 months were not significant within each subgroup (data available/data not available at 1 year), overall more missing data is present when ‘invasive’ disease-related events occur before 12 months. This is further reflected in Supplementary Table 2, where patients with incomplete resection and/or residual disease (risk factors for invasive recurrence) also had more missing data at 12 months.

**Supplementary Table 2. Baseline features of 452 patients in the HRQoL sub-study, and of patients with and without paired baseline data - one year primary endpoint BLCS score value**

|  |  | **All patients in the QoL sub-study** | | **Patients with paired data at 1 year** | | **Patients with NO paired data at 1 year** | | **p-value** |
| --- | --- | --- | --- | --- | --- | --- | --- | --- |
| **Variable** |  | **N** | **%** | **N** | **%** | **N** | **%** |  |
| **Total** |  | 452 | 100.0% | 227 | 100.0% | 225 | 100.0% |  |
| **Sex** | Male | 367 | 81.2% | 187 | 82.4% | 180 | 80.0% | 0.5 |
|  | Female | 85 | 18.8% | 40 | 17.6% | 45 | 20.0% |  |
| **Age at randomisation** | Median (IQR) | 72.9 | (65.6, 77.6) | 73.2 | (65.2, 78.2) | 72.7 | (65.8, 77.0) | 0.4 |
| **WHO Performance Status** | 0 | 264 | 58.4% | 143 | 63.0% | 121 | 53.8% | 0.13 |
|  | 1 | 162 | 35.8% | 73 | 32.2% | 89 | 39.6% |  |
|  | 2 | 26 | 5.8% | 11 | 4.8% | 15 | 6.7% |  |
| **Stage** | T1 | 1 | 0.2% | 1 | 0.4% | 0 | 0.0% | 0.19 |
|  | T2 | 378 | 83.6% | 193 | 85.0% | 185 | 82.2% |  |
|  | T3a | 29 | 6.4% | 16 | 7.0% | 13 | 5.8% |  |
|  | T3b | 30 | 6.6% | 14 | 6.2% | 16 | 7.1% |  |
|  | T4a | 14 | 3.1% | 3 | 1.3% | 11 | 4.9% |  |
| **Grade** | G1 | 1 | 0.2% | 1 | 0.4% | 0 | 0.0% | 0.17 |
|  | G2 | 57 | 12.6% | 29 | 12.8% | 28 | 12.4% |  |
|  | G3 | 390 | 86.3% | 197 | 86.8% | 193 | 85.8% |  |
|  | Missing | 4 | 0.9% | 0 | 0.0% | 4 | 1.8% |  |
| **Multiple tumours** | 1 | 377 | 83.4% | 186 | 81.9% | 33 | 14.7% | 0.7 |
|  | >1 | 73 | 16.2% | 40 | 17.6% | 191 | 84.9% |  |
|  | Missing | 2 | 0.4% | 1 | 0.4% | 1 | 0.4% |  |
| **Tumour size** | <30mm | 97 | 21.5% | 55 | 24.2% | 42 | 18.7% | 0.3 |
|  | >=30mm | 196 | 43.4% | 98 | 43.2% | 98 | 43.6% |  |
|  | Missing | 159 | 35.2% | 74 | 32.6% | 85 | 37.8% |  |
| **Tumour resection** | Biopsy/Not resected | 45 | 10.0% | 17 | 7.5% | 28 | 12.4% | 0.010 |
|  | Complete Resection | 255 | 56.4% | 147 | 64.8% | 108 | 48.0% |  |
|  | Incomplete Resection | 145 | 32.1% | 60 | 26.4% | 85 | 37.8% |  |
|  | Resected (extent unknown) | 3 | 0.7% | 1 | 0.4% | 2 | 0.9% |  |
|  | Unknown | 4 | 0.9% | 2 | 0.9% | 2 | 0.9% |  |
| **Residual mass post resection** | Yes | 123 | 27.2% | 50 | 22.0% | 73 | 32.4% | 0.008 |
|  | No | 304 | 67.3% | 168 | 74.0% | 136 | 60.4% |  |
|  | Unknown | 25 | 5.5% | 9 | 4.0% | 16 | 7.1% |  |
| **Neoadjuvant CT planned** | No | 319 | 70.6% | 160 | 70.5% | 159 | 70.7% | >0.9 |
|  | Yes | 133 | 29.4% | 67 | 29.5% | 66 | 29.3% |  |
| **Planned RT schedule** | 55Gy/20F | 177 | 39.2% | 80 | 35.2% | 97 | 43.1% | 0.087 |
|  | 64Gy/32F | 275 | 60.8% | 147 | 64.8% | 128 | 56.9% |  |
| **RT randomisation** | stRT | 107 | 23.7% | 52 | 22.9% | 55 | 24.4% | 0.7 |
|  | RHDVRT | 109 | 24.1% | 52 | 22.9% | 57 | 25.3% |  |
|  | Elect stRT | 236 | 52.2% | 123 | 54.2% | 113 | 50.2% |  |
| **CT randomisation** | CT | 179 | 39.6% | 89 | 39.2% | 90 | 40.0% | >0.9 |
|  | No CT | 176 | 38.9% | 88 | 38.8% | 88 | 39.1% |  |
|  | Elect no CT | 97 | 21.5% | 50 | 22.0% | 47 | 20.9% |  |

Q1: 1st quartile (25% percentile), Q3: 3^rd^ quartile (75% percentile)

**Supplementary Figure 3. Mean changes from baseline, other FACT subscales - overall population**

*Paired t-test p-value <0.01

**Supplementary Figure 4. FACT-BL Changes from baseline, CT comparison**

**Supplementary Figure 5. FACT-BL Changes from baseline, RT comparison**

**Supplementary Table 3**. FACT-BL TOTAL and BLCS scores per timepoint – by neoadjuvant use

|  | Baseline | | | EOT | | | 1 year | | | 5 years | | |
| --- | --- | --- | --- | --- | --- | --- | --- | --- | --- | --- | --- | --- |
|  | N | Median | Q1-Q3 | N | Median | Q1-Q3 | N | Median | Q1-Q3 | N | Median | Q1-Q3 |
| **No neoadjuvant** |  |  |  |  |  |  |  |  |  |  |  |  |
| BLCS | 294 | 34 | 29-38 | 251 | 29 | 23-34 | 171 | 35 | 31-38 | 81 | 34 | 30-37 |
| TOTAL | 293 | 123 | 107-133 | 251 | 114 | 97-130 | 170 | 126 | 107-137 | 79 | 125 | 115-135 |
| **Neoadjuvant** |  |  |  |  |  |  |  |  |  |  |  |  |
| BLCS | 127 | 36 | 31-40 | 98 | 30 | 24-35 | 71 | 35 | 30-40 | 28 | 36 | 33-41 |
| TOTAL | 128 | 125 | 111-135 | 98 | 117 | 102-131 | 70 | 126 | 118-138 | 28 | 130 | 116-143 |

**Supplementary Table 4**. Change from baseline in TOTAL and BLCS scores at one year by neoadjuvant use

|  | Neoadjuvant | | | No neoadjuvant | | | Difference Neoadjuvant Yes-No§ | | |
| --- | --- | --- | --- | --- | --- | --- | --- | --- | --- |
| 1 year | N | Mean | 99% CI | N | Mean | 99% CI | Mean† | 99% CI | p-value* |
| BLCS | 67 | -0.14 | -2.28, 2.01 | 160 | -0.39 | -1.80, 1.02 | 0.93 | -1.39, 3.25 | 0.3 |
| TOTAL | 67 | 5.62 | -0.51, 11.75 | 159 | -0.06 | -3.80, 3.67 | 6.17 | -0.50, 12.85 | 0.017 |
| §Positive differences favour neoadjuvant group | | | |  |  |  |  |  |  |
| †Mean difference between groups, computed by ANCOVA and adjusted by CT randomisation, RT randomisation, radiotherapy fractionation schedule and baseline score. | | | | | | | | | |
| *To account for multiplicity, significance at p-value<0.01 | | | |  |  |  |  |  |  |
| BLCS= Bladder cancer subscale | | | | | | | | | |

**Supplementary Figure 6**. Mean change from baseline (with 99% confidence intervals) in BLCS and TOTAL scores by neoadjuvant use

| **** | **** |
| --- | --- |

**Supplementary Figure 7. Individual BLCS items per randomised comparisons**

**Supplementary Table 5. Clinically relevant change from baseline score for the BLCS subscale, by timepoint (CT comparison).**

| **Timepoint** |  | **Chemotherapy** | | **No Chemotherapy** | | **P value** |
| --- | --- | --- | --- | --- | --- | --- |
|  | **Change from baseline** | **N** | **%** | **N** | **%** |  |
| **End of treatment** | Worsening (≤ - 3) | 76 | 56.3% | 73 | 57.5% | 0.085 |
|  | No difference (-2, 2) | 37 | 27.4% | 44 | 34.6% |  |
|  | Improvement (≥ +3) | 22 | 16.3% | 10 | 7.9% |  |
| **6 months** | Worsening (≤ - 3) | 38 | 33.3% | 39 | 37.1% | 0.4 |
|  | No difference (-2, 2) | 38 | 33.3% | 40 | 38.1% |  |
|  | Improvement (≥ +3) | 38 | 33.3% | 26 | 24.8% |  |
| **12 months** | Worsening (≤ - 3) | 31 | 34.8% | 33 | 37.5% | 0.5 |
|  | No difference (-2, 2) | 38 | 42.7% | 30 | 34.1% |  |
|  | Improvement (≥ +3) | 20 | 22.5% | 25 | 28.4% |  |
| **2 years** | Worsening (≤ - 3) | 31 | 47.0% | 21 | 30.0% | 0.033 |
|  | No difference (-2, 2) | 15 | 22.7% | 30 | 42.9% |  |
|  | Improvement (≥ +3) | 20 | 30.3% | 19 | 27.1% |  |
| **3 years** | Worsening (≤ - 3) | 18 | 28.6% | 15 | 29.4% | 0.9 |
|  | No difference (-2, 2) | 27 | 42.9% | 23 | 45.1% |  |
|  | Improvement (≥ +3) | 18 | 28.6% | 13 | 25.5% |  |
| **4 years** | Worsening (≤ - 3) | 19 | 34.5% | 10 | 22.2% | 0.3 |
|  | No difference (-2, 2) | 23 | 41.8% | 25 | 55.6% |  |
|  | Improvement (≥ +3) | 13 | 23.6% | 10 | 22.2% |  |
| **5 years** | Worsening (≤ - 3) | 22 | 41.5% | 11 | 33.3% | 0.7 |
|  | No difference (-2, 2) | 20 | 37.7% | 15 | 45.5% |  |
|  | Improvement (≥ +3) | 11 | 20.8% | 7 | 21.2% |  |

**Supplementary Table 6. Clinically relevant change from baseline score for the TOI scale, by timepoint (CT comparison).**

| **Timepoint** |  | **Chemotherapy** | | **No Chemotherapy** | | **P value** |
| --- | --- | --- | --- | --- | --- | --- |
|  | **Change from baseline** | **N** | **%** | **N** | **%** |  |
| **End of treatment** | Worsening (≤ - 5) | 74 | 56.9% | 80 | 63.0% | 0.3 |
|  | No difference (-4, 4) | 32 | 24.6% | 32 | 25.2% |  |
|  | Improvement (≥ +5) | 24 | 18.5% | 15 | 11.8% |  |
| **6 months** | Worsening (≤ - 5) | 31 | 27.4% | 39 | 37.1% | 0.2 |
|  | No difference (-4, 4) | 38 | 33.6% | 35 | 33.3% |  |
|  | Improvement (≥ +5) | 44 | 38.9% | 31 | 29.5% |  |
| **12 months** | Worsening (≤ - 5) | 28 | 31.8% | 30 | 34.9% | 0.9 |
|  | No difference (-4, 4) | 27 | 30.7% | 26 | 30.2% |  |
|  | Improvement (≥ +5) | 33 | 37.5% | 30 | 34.9% |  |
| **2 years** | Worsening (≤ - 5) | 22 | 34.4% | 17 | 24.6% | 0.5 |
|  | No difference (-4, 4) | 21 | 32.8% | 27 | 39.1% |  |
|  | Improvement (≥ +5) | 21 | 32.8% | 25 | 36.2% |  |
| **3 years** | Worsening (≤ - 5) | 21 | 33.9% | 15 | 30.0% | 0.5 |
|  | No difference (-4, 4) | 18 | 29.0% | 20 | 40.0% |  |
|  | Improvement (≥ +5) | 23 | 37.1% | 15 | 30.0% |  |
| **4 years** | Worsening (≤ - 5) | 14 | 25.9% | 9 | 20.9% | 0.5 |
|  | No difference (-4, 4) | 25 | 46.3% | 17 | 39.5% |  |
|  | Improvement (≥ +5) | 15 | 27.8% | 17 | 39.5% |  |
| **5 years** | Worsening (≤ - 5) | 19 | 36.5% | 8 | 24.2% | 0.5 |
|  | No difference (-4, 4) | 18 | 34.6% | 15 | 45.5% |  |
|  | Improvement (≥ +5) | 15 | 28.8% | 10 | 30.3% |  |

**Supplementary Table 7. Clinically relevant change from baseline score for the TOTAL FACT-BL questionnaire, by timepoint (CT comparison).**

| **Timepoint** |  | **Chemotherapy** | | **No Chemotherapy** | | **p-value** |
| --- | --- | --- | --- | --- | --- | --- |
|  | **Change from baseline** | **N** | **%** | **N** | **%** |  |
| **End of treatment** | Worsening (≤ - 7) | 70 | 53.0% | 63 | 48.8% | 0.085 |
|  | No difference (-6, 6) | 35 | 26.5% | 49 | 38.0% |  |
|  | Improvement (≥ +7) | 27 | 20.5% | 17 | 13.2% |  |
| **6 months** | Worsening (≤ - 7) | 33 | 28.7% | 36 | 34.0% | 0.3 |
|  | No difference (-6, 6) | 38 | 33.0% | 40 | 37.7% |  |
|  | Improvement (≥ +7) | 44 | 38.3% | 30 | 28.3% |  |
| **12 months** | Worsening (≤ - 7) | 30 | 33.7% | 23 | 26.7% | 0.6 |
|  | No difference (-6, 6) | 27 | 30.3% | 31 | 36.0% |  |
|  | Improvement (≥ +7) | 32 | 36.0% | 32 | 37.2% |  |
| **2 years** | Worsening (≤ - 7) | 18 | 28.1% | 18 | 25.7% | 0.7 |
|  | No difference (-6, 6) | 22 | 34.4% | 29 | 41.4% |  |
|  | Improvement (≥ +7) | 24 | 37.5% | 23 | 32.9% |  |
| **3 years** | Worsening (≤ - 7) | 15 | 24.2% | 16 | 31.4% | 0.7 |
|  | No difference (-6, 6) | 21 | 33.9% | 17 | 33.3% |  |
|  | Improvement (≥ +7) | 26 | 41.9% | 18 | 35.3% |  |
| **4 years** | Worsening (≤ - 7) | 11 | 20.4% | 8 | 18.6% | 0.9 |
|  | No difference (-6, 6) | 27 | 50.0% | 20 | 46.5% |  |
|  | Improvement (≥ +7) | 16 | 29.6% | 15 | 34.9% |  |
| **5 years** | Worsening (≤ - 7) | 16 | 30.8% | 9 | 27.3% | 0.4 |
|  | No difference (-6, 6) | 19 | 36.5% | 17 | 51.5% |  |
|  | Improvement (≥ +7) | 17 | 32.7% | 7 | 21.2% |  |

**Supplementary Table 8. Clinically relevant change from baseline score for the BLCS subscale, by timepoint (RT comparison).**

| **Timepoint** |  | **Standard radiotherapy** | | **Reduced high dose volume RT** | | **p-value** |
| --- | --- | --- | --- | --- | --- | --- |
|  | **Change from baseline** | **N** | **%** | **N** | **%** |  |
| **End of treatment** | Worsening (≤ - 3) | 35 | 53.0% | 51 | 64.6% | 0.3 |
|  | No difference (-2, 2) | 22 | 33.3% | 18 | 22.8% |  |
|  | Improvement (≥ +3) | 9 | 13.6% | 10 | 12.7% |  |
| **6 months** | Worsening (≤ - 3) | 19 | 29.7% | 17 | 28.3% | >0.9 |
|  | No difference (-2, 2) | 22 | 34.4% | 22 | 36.7% |  |
|  | Improvement (≥ +3) | 23 | 35.9% | 21 | 35.0% |  |
| **12 months** | Worsening (≤ - 3) | 14 | 26.9% | 12 | 23.1% | 0.8 |
|  | No difference (-2, 2) | 23 | 44.2% | 26 | 50.0% |  |
|  | Improvement (≥ +3) | 15 | 28.8% | 14 | 26.9% |  |
| **2 years** | Worsening (≤ - 3) | 10 | 26.3% | 12 | 33.3% | 0.6 |
|  | No difference (-2, 2) | 11 | 28.9% | 12 | 33.3% |  |
|  | Improvement (≥ +3) | 17 | 44.7% | 12 | 33.3% |  |
| **3 years** | Worsening (≤ - 3) | 10 | 31.3% | 13 | 35.1% | 0.8 |
|  | No difference (-2, 2) | 10 | 31.3% | 13 | 35.1% |  |
|  | Improvement (≥ +3) | 12 | 37.5% | 11 | 29.7% |  |
| **4 years** | Worsening (≤ - 3) | 9 | 32.1% | 7 | 25.9% | 0.3 |
|  | No difference (-2, 2) | 8 | 28.6% | 13 | 48.1% |  |
|  | Improvement (≥ +3) | 11 | 39.3% | 7 | 25.9% |  |
| **5 years** | Worsening (≤ - 3) | 6 | 26.1% | 11 | 45.8% | 0.4* |
|  | No difference (-2, 2) | 11 | 47.8% | 8 | 33.3% |  |
|  | Improvement (≥ +3) | 6 | 26.1% | 5 | 20.8% |  |

*Fishers exact test used owing to small cell frequencies.

**Supplementary Table 9. Clinically relevant change from baseline score for the TOI scale, by timepoint (RT comparison).**

| **Timepoint** |  | **Standard radiotherapy** | | **Reduced high dose volume RT** | | **p-value** |
| --- | --- | --- | --- | --- | --- | --- |
|  | **Change from baseline** | **N** | **%** | **N** | **%** |  |
| **End of treatment** | Worsening (≤ - 5) | 33 | 50.8% | 48 | 61.5% | 0.19 |
|  | No difference (-4, 4) | 24 | 36.9% | 18 | 23.1% |  |
|  | Improvement (≥ +5) | 8 | 12.3% | 12 | 15.4% |  |
| **6 months** | Worsening (≤ - 5) | 16 | 25.4% | 16 | 27.1% | 0.5 |
|  | No difference (-4, 4) | 18 | 28.6% | 22 | 37.3% |  |
|  | Improvement (≥ +5) | 29 | 46.0% | 21 | 35.6% |  |
| **12 months** | Worsening (≤ - 5) | 16 | 30.8% | 13 | 25.0% | 0.4 |
|  | No difference (-4, 4) | 13 | 25.0% | 19 | 36.5% |  |
|  | Improvement (≥ +5) | 23 | 44.2% | 20 | 38.5% |  |
| **2 years** | Worsening (≤ - 5) | 9 | 24.3% | 13 | 36.1% | 0.6* |
|  | No difference (-4, 4) | 13 | 35.1% | 11 | 30.6% |  |
|  | Improvement (≥ +5) | 15 | 40.5% | 12 | 33.3% |  |
| **3 years** | Worsening (≤ - 5) | 9 | 28.1% | 16 | 44.4% | 0.3* |
|  | No difference (-4, 4) | 11 | 34.4% | 11 | 30.6% |  |
|  | Improvement (≥ +5) | 12 | 37.5% | 9 | 25.0% |  |
| **4 years** | Worsening (≤ - 5) | 8 | 29.6% | 9 | 34.6% | 0.8* |
|  | No difference (-4, 4) | 12 | 44.4% | 9 | 34.6% |  |
|  | Improvement (≥ +5) | 7 | 25.9% | 8 | 30.8% |  |
| **5 years** | Worsening (≤ - 5) | 7 | 31.8% | 8 | 33.3% | >0.9* |
|  | No difference (-4, 4) | 9 | 40.9% | 9 | 37.5% |  |
|  | Improvement (≥ +5) | 6 | 27.3% | 7 | 29.2% |  |

*Fishers exact test used owing to small cell frequencies

**Supplementary Table 10. Clinically relevant change from baseline score for the TOTAL FACT-BL questionnaire, by timepoint (RT comparison).**

| **Timepoint** |  | **Standard radiotherapy** | | **Reduced high dose volume RT** | | **p-value** |
| --- | --- | --- | --- | --- | --- | --- |
|  | **Change from baseline** | **N** | **%** | **N** | **%** |  |
| **End of treatment** | Worsening (≤ - 7) | 24 | 35.8% | 40 | 51.3% | 0.15 |
|  | No difference (-6, 6) | 28 | 41.8% | 27 | 34.6% |  |
|  | Improvement (≥ +7) | 15 | 22.4% | 11 | 14.1% |  |
| **6 months** | Worsening (≤ - 7) | 16 | 25.0% | 14 | 23.0% | 0.8 |
|  | No difference (-6, 6) | 22 | 34.4% | 24 | 39.3% |  |
|  | Improvement (≥ +7) | 26 | 40.6% | 23 | 37.7% |  |
| **12 months** | Worsening (≤ - 7) | 17 | 32.1% | 11 | 20.8% | 0.4 |
|  | No difference (-6, 6) | 15 | 28.3% | 18 | 34.0% |  |
|  | Improvement (≥ +7) | 21 | 39.6% | 24 | 45.3% |  |
| **2 years** | Worsening (≤ - 7) | 8 | 21.6% | 11 | 29.7% | 0.6* |
|  | No difference (-6, 6) | 11 | 29.7% | 12 | 32.4% |  |
|  | Improvement (≥ +7) | 18 | 48.6% | 14 | 37.8% |  |
| **3 years** | Worsening (≤ - 7) | 7 | 21.9% | 10 | 27.0% | 0.9* |
|  | No difference (-6, 6) | 10 | 31.3% | 12 | 32.4% |  |
|  | Improvement (≥ +7) | 15 | 46.9% | 15 | 40.5% |  |
| **4 years** | Worsening (≤ - 7) | 6 | 22.2% | 8 | 29.6% | 0.7* |
|  | No difference (-6, 6) | 10 | 37.0% | 8 | 29.6% |  |
|  | Improvement (≥ +7) | 11 | 40.7% | 11 | 40.7% |  |
| **5 years** | Worsening (≤ - 7) | 2 | 9.1% | 8 | 33.3% | 0.14* |
|  | No difference (-6, 6) | 12 | 54.5% | 9 | 37.5% |  |
|  | Improvement (≥ +7) | 8 | 36.4% | 7 | 29.2% |  |

*Fishers exact test used owing to small cell frequencies
